# The Gene Expression Response of Chronic Lymphocytic Leukemia Cells to IL-4 Is Specific, Depends on ZAP-70 Status and Is Differentially Affected by an NFκB Inhibitor

**DOI:** 10.1371/journal.pone.0109533

**Published:** 2014-10-03

**Authors:** Natalia Ruiz-Lafuente, María-José Alcaraz-García, Silvia Sebastián-Ruiz, Joaquín Gómez-Espuch, Consuelo Funes, José-María Moraleda, María-Carmen García-Garay, Natividad Montes-Barqueros, Alfredo Minguela, María-Rocío Álvarez-López, Antonio Parrado

**Affiliations:** 1 Servicio de Inmunología, Hospital Clínico Universitario Virgen de la Arrixaca, Murcia, Spain; 2 Servicio de Hematología y Hemoterapia, Hospital Clínico Universitario Virgen de la Arrixaca, Murcia, Spain; 3 Servicio de Hematología y Hemoterapia, Hospital General Universitario Santa Lucía, Cartagena, Spain; 4 Universidad de Murcia, Murcia, Spain; 5 Centro de Investigación Biomédica en Red de enfermedades hepáticas y digestivas, Murcia, Spain; 6 Instituto Murciano de Investigación Biosanitaria Virgen de la Arrixaca (IMIB-Arrixaca), Murcia, Spain; Queen's University Belfast, United Kingdom

## Abstract

Interleukin 4 (IL-4), an essential mediator of B cell development, plays a role in survival of chronic lymphocytic leukemia (CLL) cells. To obtain new insights into the function of the IL-4 pathway in CLL, we analyzed the gene expression response to IL-4 in CLL and in normal B cells (NBC) by oligonucleotide microarrays, resulting in the identification of 232 non-redundant entities in CLL and 146 in NBC (95 common, 283 altogether), of which 189 were well-defined genes in CLL and 123 in NBC (83 common, 229 altogether) (p<0.05, 2-fold cut-off). To the best of our knowledge, most of them were novel IL-4 targets for CLL (98%), B cells of any source (83%), or any cell type (70%). Responses were significantly higher for 54 and 11 genes in CLL and NBC compared to each other, respectively. In CLL, ZAP-70 status had an impact on IL-4 response, since different sets of IL-4 targets correlated positively or negatively with baseline expression of ZAP-70. In addition, the NFκB inhibitor 6-Amino-4-(4-phenoxyphenethylamino)quinazoline, which reversed the anti-apoptotic effect of IL-4, preferentially blocked the response of genes positively correlated with ZAP-70 (e.g. CCR2, SUSD2), but enhanced the response of genes negatively correlated with ZAP-70 (e.g. AUH, BCL6, LY75, NFIL3). Dissection of the gene expression response to IL-4 in CLL and NBC contributes to the understanding of the anti-apoptotic response. Initial evidence of a connection between ZAP-70 and NFκB supports further exploration of targeting NFκB in the context of the assessment of inhibition of the IL-4 pathway as a therapeutic strategy in CLL, especially in patients expressing bad prognostic markers.

## Introduction

Chronic lymphocytic leukemia (CLL) is a malignant disease characterized by the proliferation of CD5+CD23+ B cells. The clinical course is heterogeneous in CLL. About half of patients live for decades and never require treatment, while the other half become symptomatic or progress to late stages of the disease and require chemotherapy. Low rate of mutation of the IGHV sequence, and high levels of expression of ZAP-70, CD38, and CD49d/ITGA4, are prognostic risk markers [1]. Despite this heterogeneity, gene expression profiles (GEP) in CLL are relatively homogeneous, considering that specific CLL signatures clearly discriminate CLL cells from B cells of other related pathologic entities and from normal B cells [2]–[8], whereas specific signatures for CLL prognostic groups are based on more subtle differences [9]–[12]. CLL cells spontaneously and rapidly die *in vitro*, because they lack essential signals provided by the natural microenvironment [13]. CLL cells interact with bone marrow stromal cells, and with T cells, antigen-presenting cells and dendritic cells within the lymph node proliferation centers (or pseudofollicles). Cytokines, chemokines, integrins, and other ligands and receptors play key roles in proliferation and survival within these cellular niches [14].

Interleukin-4 (IL-4) is a cytokine secreted by activated T cells, NK-T cells, basophils, eosinophils and mast cells. Paracrine stimulation through the IL-4 membrane receptor (IL-4R) induces signaling cascades leading to maturation of B-cell precursors into immunoglobulin-secreting cells and antigen presenting cells, proliferation of activated B cells, and induction of isotype switching toward IgE [15]. The activated IL-4R phosphorylates JAK1 and JAK3. JAK1 phosphorylates STAT6 which homodimerizes and enter the nucleus to regulate gene expression. JAK1 and JAK3 lead to anti-apoptotic signaling through PI3K/AKT and the mitochondrial pathway, and through the Ras/MAPK pathway and NFκB activation [16]. NFκB activation is anti-apoptotic in CLL [17], [18]. In B cells, IL-4 induces preferentially the non-canonical NFκB pathway [19]. IL-4 induces efficient STAT6 phosphorylation and activation in CLL [20]. However, binding of NFκB to the promoter of IGHE, CD86 and MHCII is necessary for STAT6 binding and transcription [19], [21], [22].

IL-4 efficiently protects CLL cells from spontaneous apoptosis or killing with agents such as fludarabine and chlorambucil [13], [24], [25]. CLL cells have been reported to be more prone than normal B cells (NBC) to spontaneous apoptosis [13], and those expressing good prognostic markers more than those expressing bad prognostic markers [23]. IL-4 acts in a paracrine rather than autocrine manner in CLL [26]. GEPs in follicular lymphoma suggest that a connection dependent on IL-4 between T cells and the malignant B cells sustains tumorigenesis [27]. Similarly, IL-4 could play a role in CLL pathogenesis and progression. Several studies have focused on identifying the IL-4 targets in mouse B splenocytes [28], some lymphoma subtypes [29], and other non B cell types (see additional references in Table S5). However, the gene expression response to IL-4 in CLL is poorly known.

We report here the first study aimed at identifying the IL-4 targets in CLL. We found sets of genes differentially regulated by IL-4 in CLL and NBC, and within CLL, depending on ZAP-70 expression, suggesting that the gene expression response to IL-4 may be relevant in CLL pathogenesis and prognosis. Finally, we found evidence for a dual mechanism which links the gene expression response to IL-4, NFκB activity, and ZAP-70 expression, based on the observation that a proportion of the IL-4 targets have a higher response in ZAP-70 positive patients which can be blocked by an NFκB inhibitor, and another group of IL-4 targets have a higher response in ZAP-70 negative patients which can be further induced by the NFκB inhibitor.

## Methods

### Sample collection

Peripheral blood samples from 38 chronic lymphocytic leukemia (CLL) patients and 13 controls with normal lymphopoiesis were obtained. The study was approved by the Review Board of Hospital Clínico Universitario Virgen de la Arrixaca, and the participants provided their written informed consent. All the patients had leukocytosis and did not receive treatment during the prior 3 months to sample collection. From the 38 CLL patients, 23 were studied by microarray and 15 were included later to increase the statistical significance of validations (Table S1).

### Cell isolation

Samples were processed to isolate the B cells by negative selection procedures which were based on cocktails containing CD2, CD16, CD36 and CD235a antibodies for depletion of T cells, NK cells, monocytes, macrophages, and erythrocytes. The RossetteSep Human B Cell Enrichment Cocktail kit (StemCell Technologies, Vancouver, Canada) was suitable for CLL samples, since these are rich in malignant B cells. Small volumes of peripheral blood (10 mL) were collected from patients, and B cell isolation was directly performed during Ficoll centrifugation, following the manufacturer's instructions. The Dynabeads Untouched Human B cells kit (Invitrogen, Carlsbad, CA) was a suitable choice for normal B cells (NBC) due to the low content of B cells in the peripheral blood of normal subjects (usually less than 10% of the lymphocytes). Larger volumes of peripheral blood were collected (500 mL), PBMC were isolated by centrifugation over Ficoll 1.077 g/mL, and NBC isolated using the kit, following the manufacturer's instructions. Enrichment was determined by labelling with CD19-FITC, CD3-PE, and CD5-PE-Cy7 ((BD Biosciences), followed by acquisition in a FACScalibur flow cytometer (BD Biosciences), and analysis using the CellQuest software. Purity of CD19+ cells was 93.5±1.41% (mean ±s.e.m.) in NBC and 97.54±0.34% in CLL, including 0.56±0.17% of CD19+CD5- potential normal B cells within the CLL fractions (range 0–1.98%). The percentage of ZAP-70 positive cells within the CD19+CD5+ fraction of CLL was determined from aliquots of peripheral blood subjected to red cell lysis, permeabilization with the Cytofix/Cytoperm kit, and labelling with CD19-FITC, ZAP70-PE, CD5-PE-Cy7, and CD3-APC (BD Biosciences).

### Cell culture and determination of apoptosis

Following purification, three fractions of the purified CLL and NBC were processed: a) at time zero (“Pre”); b) after being cultured for 18 hours in RPMI-1640 medium supplemented with 10% fetal calf serum (Cambrex, East Rutherford, NJ) (“Ctrl”); and c) as b, but with adding 10 ng/mL of human recombinant IL-4 (BD Biosciences, San Diego, CA) (“IL-4”). In the absence of sufficient material, the Ctrl culture was not carried out in 3 NBC (NBC06, NBC07, and NBC08). In selected patients, additional fractions were treated with IL-4 plus InSolution NF-kB activation inhibitor [6-Amino-4-(4-phenoxyphenylethylamino)quinazoline] (Merck, Nottingham, UK) at 1 µM and 10 µM. Apoptosis of the cultured cells was determined by dual labelling with annexin V and propidium iodide (BD Biosciences), and flow cytometry analysis.

### RNA isolation

Total RNA was isolated using the miRNeasy Mini Kit (Qiagen, Hilden, Germany). RNA samples were quantitated in a NanoDrop 2000 (Thermo Fisher Scientific, Whaltham, MA). RNA quality was examined in an Agilent 2100 Bioanalyzer (Agilent Technologies, Palo Alto, CA). Only samples with R.I.N. (RNA Integrity Number) >7.0 were further studied.

### Microarray analysis

**Figure 1:**
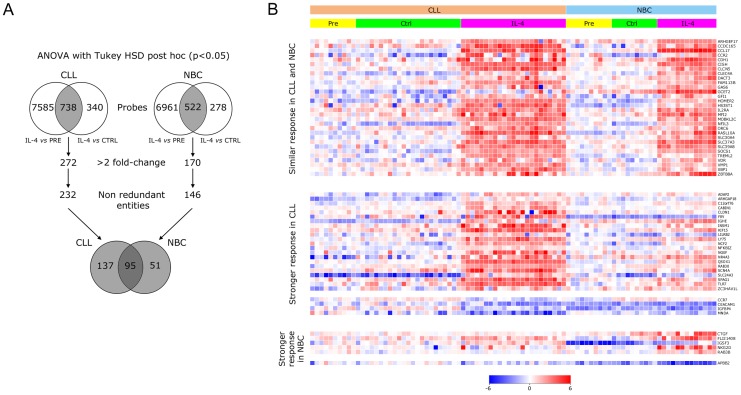
Identification of the IL-4 targets in CLL and in NBC. (A) Scheme of the strategy used to identify the IL-4 targets in patients and in controls. (B) Heat maps for expression of IL-4 targets which had: *top panel*, similar changes in CLL and NBC and above 3-fold change for both; *center panel*, significantly higher changes in CLL and above 3-fold change in CLL; *bottom panel*, significantly higher changes in NBC and above 3-fold change in NBC. IL-4 targets are ordered alphabetically. In the event that several probes represent the same gene, only one is shown. Relative expression levels are depicted according to the shown log_2_ color scale.

From each RNA sample, 250 ng were labeled with cyanine 5-CTP (Cy5) using Agilent Two Color Quick Amp Labeling and RNA Spike-In kits, according to the manufacturer's protocol. A pooled sample composed of equimolar amounts of RNA from Pre samples of 4 CLL and 4 NBC (reference), was labelled with cyanine 3-CTP (Cy3). The labeled cRNAs were mixed together and hybridized onto Agilent Whole Human Genome Microarrays (4×44 k) targeting 19,596 Entrez Gene RNAs, using the Agilent Gene Expression Hybridization kit. After hybridization, the microarray slides were washed and scanned in an Agilent G2565CA DNA Microarray Scanner. Images were analyzed with the Agilent Feature Extraction software to automatically generate the datasets. Log_10_ ratios (test vs reference) were computed after normalization correction performed by linear and Lowess methods. The datasets were statistically analyzed and visualized using the GeneSpring GX software (Agilent), using the one-way ANOVA test (p<0.05) between samples Pre, Ctrl, and IL-4, with post hoc Tukey HSD analysis (Figure 1A). The computation of p-values was performed using the Benjamini-Hochberg FDR correction. CLL and NBC were analyzed independently for each cell type. Those entities that passed the analysis with increases or decreases above 2-fold for comparisons IL-4 vs Pre, and IL-4 vs Ctrl concurrently and with the same direction of change were considered IL-4 targets. Similarly, entities that passed the ANOVA test with post hoc analysis with increases or decreases above 2-fold for comparisons Pre vs Ctrl, and IL-4 vs Ctrl concurrently and with the same direction of change, excluding some genes that were among the IL-4 targets, represented genes whose expression was altered by cell culture but remained stable under culture with IL-4. Finally, entities that passed the ANOVA test with post hoc analysis with increases or decreases above 2-fold for comparisons Pre vs Ctrl, and Pre vs IL-4 concurrently and with the same direction of change, excluding some genes that were in the previous two lists, represented genes whose expression was altered by cell culture and not stabilized by IL-4. Our initial analysis performed with the first 10 patients and controls showed an elevated number of entities with significant changes induced by cell culture and not stabilized by IL-4, indicating that our approach of subtracting sample Ctrl was essential to identify changes specifically induced by IL-4. However, most changes identified for comparisons IL-4 vs Ctrl were also significant for comparison IL-4 vs Pre. For this reason, comparison IL-4 vs Pre was judged as dispensable from that time. Thus, our final microarray analysis was based on 23 CLL patients (10 Pre, 23 Ctrl, and 23 IL-4), and 13 NBC samples (10 Pre, 10 Ctrl, and 13 IL-4). Cells from patient CLL01 treated with IL-4 plus NFκB inhibitor was also studied by microarray. Datasets were deposited at the Gene Expression Omnibus database under accession number GSE55288. After defining the IL-4 targets, to determine whether they had responses of different magnitude between CLL and NBC, the extent of changes in each individual (expressed as log_2_ ratios for IL-4 vs Ctrl) was compared by the Student t test using the SigmaStat statistical analysis package (Systat Software Inc, San Jose, CA). In addition, the behavior of the different IL-4 targets was compared between patients by Pearson correlation analysis using SigmaStat, and by hierarchical clustering analysis using the Ward's linkage method on euclidean distances. Predictions for molecular interactions, activation state of trascription factors, biological functions, canonical pathways, and process networks, interpreted from the sets of genes differentially expressed, were performed using the MetaCore software (Thomsom Reuters Systems Biology, New York City, NY).

### Quantitative PCR (qPCR)

RNA samples were subjected to retrotranscription with the iScript cDNA Synthesis Kit (Bio-Rad, Hercules, CA), following the manufacturer's instructions. QPCR was performed with the SYBR Premix Ex Taq (Takara Bio, Mountain View, CA) in an ABI Prism 7000 Sequence Detection System. QuantiTect primer assays (Qiagen) specific for the following genes were used: AUH, BCL6, CCR2, CDH1, CLDN1, CTGF, FRY, GAPDH, GCET2, INSM1, LILRB1, LILRB2, LY75, MFI2, NFIL3, NGEF, NKG2D, RAB3B, SLC24A3, SUSD2, and ZAP-70. ZAP-70 mRNA quantification via qPCR is a strong surrogate marker of IGHV mutational status and a powerful prognostic factor [30]. This method can be applied to purified B cells, and does not require an internal control (T cells) to evaluate the positivity limit. Both qPCR and microarray measures of ZAP-70 expression were used for analysis of correlations with GEPs. GAPDH was used as reference for all the qPCR assays. Measures were performed in duplicate. Analysis with the 7000 System SDS software provided the cycle threshold (Ct) values. The average Ct values for GAPDH were subtracted from the average Ct values for IL-4 target genes, resulting in the ΔCt values. Next, the ΔCt value for the reference sample was subtracted from all the others including itself, resulting in the ΔΔCt values. Finally, the relative expression values, expressed as fold change compared to the reference, were generated using the formula 2^−ΔΔCt^. For validation of microarray data, the fold changes obtained by microarray, expressed as log_2_, and the −ΔΔCt values obtained by qPCR were compared by the Student t test, and by Pearson correlation analysis, using SigmaStat.

## Results

### Basal GEPs of CLL and NBC

To obtain an indication that our methodological approach was reliable, the baseline GEPs of CLL and NBC were compared by the Student t test, and the genes differentially expressed were contrasted with the literature [2]–[12]. Many of the genes significantly overexpressed (e.g. ABCA6, FMOD, IGFBP4, IGSF3, LEF1, RASGRF1, RHOC, ROR1, WNT3), or underexpressed (e.g. EBF1, HIF1A, IQSEC1, KLF3, MS4A1, SIPA1, TRIB2, TUBB1, VAV3, ZBTB16) in CLL, were concordant with previous studies, thereby providing proof of the validity of our microarray study. The complete list (cut-off 2-fold, p<0.05 (Table S2)), and a heat map representation of the most significant (cut-off 3-fold, p<0.001 (Figure S1)), are provided. Of note, no significant difference for IL4R expression was found between CLL and NBC.

### Identification of IL-4 targets in CLL and NBC

Microarray analysis identified 232 non-redundant entities in CLL (188 upregulated, 44 downregulated; Table S3), and 146 non-redundant entities in NBC (133 up, 13 down; Table S4) as IL-4 targets (cut-off 2-fold, p<0.05), being 95 common to both groups (90 up, 5 down), 137 restricted to CLL (98 up, 39 down), and 51 restricted to NBC (43 up, 8 down). Because an incubation period of 18 h allows regulation of direct and indirect IL-4 targets, we assume that the genes with the highest levels of change would probably be direct targets. The 50 IL-4 targets with the highest levels of change in CLL and NBC are shown in Table 1. From the 283 non-redundant entities identified as IL-4 targets in CLL and NBC altogether, 229 were well-defined genes (189 in CLL, 129 in NBC, 89 common), and the remaining corresponded to sequences not fully defined. To the best of our knowledge, 186 out of the 189 genes (98.5%) were novel IL-4 targets for CLL, 191 out of the 229 genes (83%) were novel IL-4 targets for B cells of any source, and 160 out of the 229 genes (70%), were novel IL-4 targets for any cell type (Table S5). Therefore, the vast majority of the genes identified in our study were novel IL-4 targets. The search for genes differentially regulated between CLL and NBC was refined by comparing the intensity of their changes by the Student t test (p<0.05). As a result, 54 genes (38 up, 16 down) had higher responses in CLL, and 11 genes (9 up, 2 down) had higher responses in NBC (Table S3, Table S4, and Figure 1B centre and bottom showing a selection of those that changed above 3-fold). These findings suggest that IL-4 probably induces divergent pathways in CLL and NBC.

**Table 1:** Top 50 IL4 targets in CLL and NBC.

CLL		NBC	
Gene Symbol	Fold Change^†^	Gene Symbol	Fold Change^†^
**SLC24A3**	61.79	CCL17	24.37
CCR2	34.08	CCR2	17.79
NFIL3	30.62	**NKG2D**	16.18
CCL17	19.82	NFIL3	15.33
HOMER2	18.72	CLEC4A	10.87
**QSOX1**	16.83	HOMER2	10.45
SOCS1	14.21	CCDC165	8.26
**NCF2**	13.85	CISH	7.65
RASL10A	13.56	HS3ST1	7.62
CLCN5	12.98	RASL10A	7.55
SLC39A8	12.29	SLC37A3	6.94
SLC37A3	12.00	QSOX1	6.92
**NGEF**	11.18	VDR	6.59
CISH	10.98	MFI2	6.51
**NR4A3**	10.08	SLC30A4	6.41
XBP1	9.47	PEG10	6.41
**SPAG1**	8.97	ENPP1	6.09
**KIF15**	7.57	**TMEM71**	6.06
BCL6	7.10	SLC39A8	5.91
**IGHE**	7.09	CLCN5	5.77
CCDC165	6.83	ZBTB8A	5.31
CLEC4A	6.65	**CTGF**	5.19
**SUSD2**	6.33	MOBKL2C	4.98
**TLR7**	6.23	NETO1	4.89
**SCN4A**	6.23	CDH1	4.83
MOBKL2C	6.03	GCET2	4.82
**INSM1**	5.90	MACROD2	4.76
**ADAP2**	5.89	**RAB3B**	4.60
RNF19A	5.82	FAM126A	4.33
CARD9	5.63	IL2RA	4.29
CDH1	5.62	GNG8	4.29
TREML2	5.56	IL4I1	4.25
TBC1D8	5.50	ARHGEF17	4.09
**FCRL2**	5.46	GAS6	4.00
**CLDN1**	5.45	ZNF443	3.96
**LY75**	5.40	**IGSF3**	3.94
SLC30A4	5.36	APOL6	3.93
AUH	5.11	PALLD	3.83
MFI2	5.08	SPINT2	3.81
GFI1	5.08	**FLJ21408**	3.79
SLC47A1	4.90	TREML2	3.76
VMP1	4.58	SOCS1	3.74
FAM126A	4.55	GFI1	3.61
CHSY1	4.53	PHF20L1	3.57
HS3ST1	4.46	KMO	3.43
GCET2	4.38	C16orf87	3.36
**ZC3HAV1L**	4.31	IGHE	3.31
RNF125	4.16	IL4R	3.30
MNDA	−4.39	**APBB2**	−3.51
CCR7	−4.48	SLC2A5	−4.07

(†) Mean fold change for comparison IL-4 vs Ctrl.

Genes in **bold** characters: significantly higher in CLL or NBC for both comparisons (IL-4 vs Pre and IL-4 vs Ctrl).

### Validation of microarray analysis

**Figure 2:**
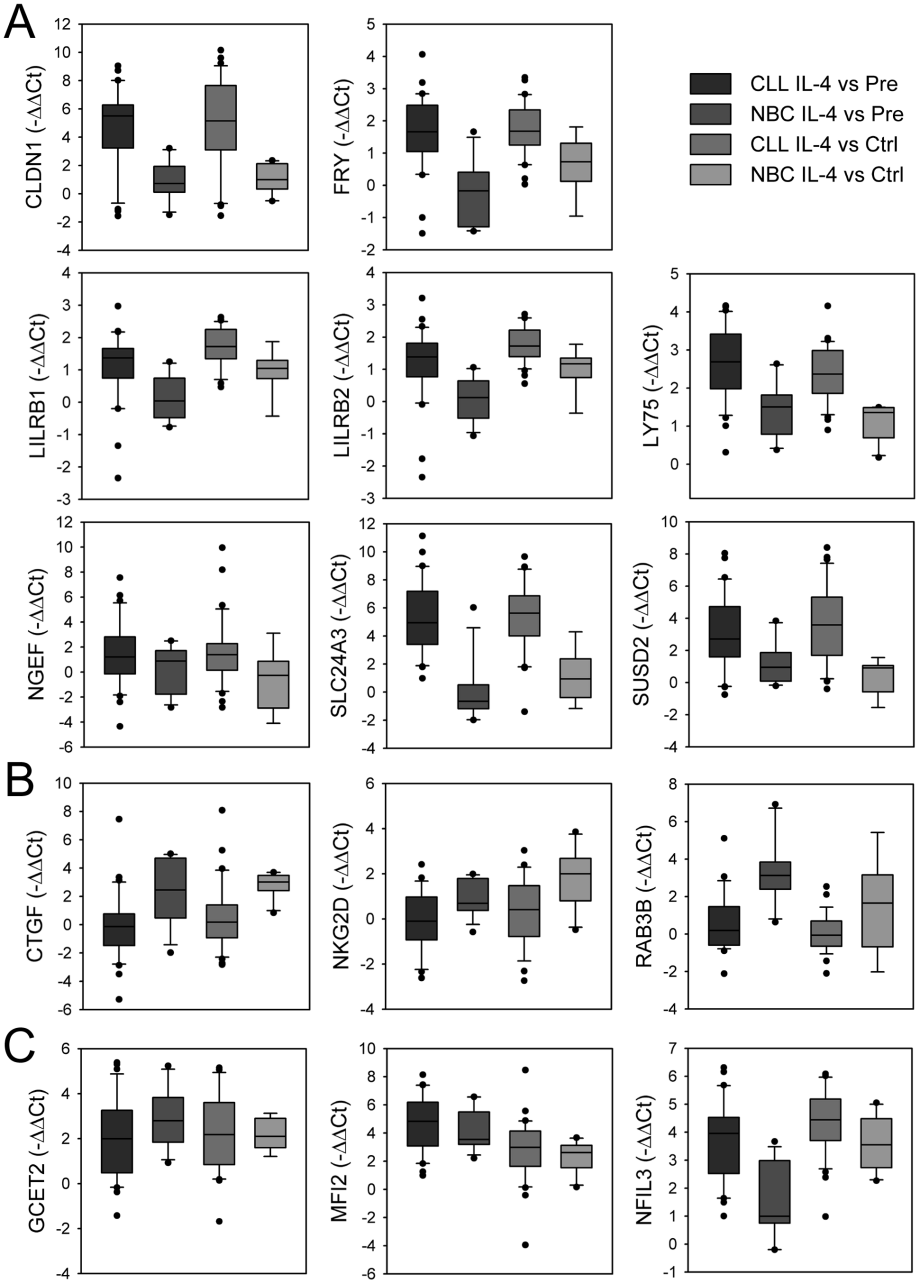
Validation of IL-4 targets in CLL and NBC by qPCR. Box whiskers representations of qPCR validations of 14 IL-4 targets representative of (A) restricted to CLL; (B) restricted to NBC; and (C) common to CLL and NBC. QPCR data are expressed as −ΔΔCt. IL-4 targets are ordered alphabetically.

To validate microarray experiments, representative IL-4 targets, restricted to CLL or NBC, or unrestricted, were assayed by qPCR (Figure 2). In most cases, comparison using the Student's t test validated microarray data. For most genes, both techniques correlated significantly (p<0.05, Figure S2). At the protein level, other authors and ourselves have provided, in previous reports, validation for several IL-4 targets detected in this study, such as CYSLTR1 [31], IGHE [32] and NFIL3 [33] in B cells, or for DOCK10 in CLL and NBC cells [34].

### Correlations between IL-4 targets, and with basal expression of ZAP-70

**Figure 3:**
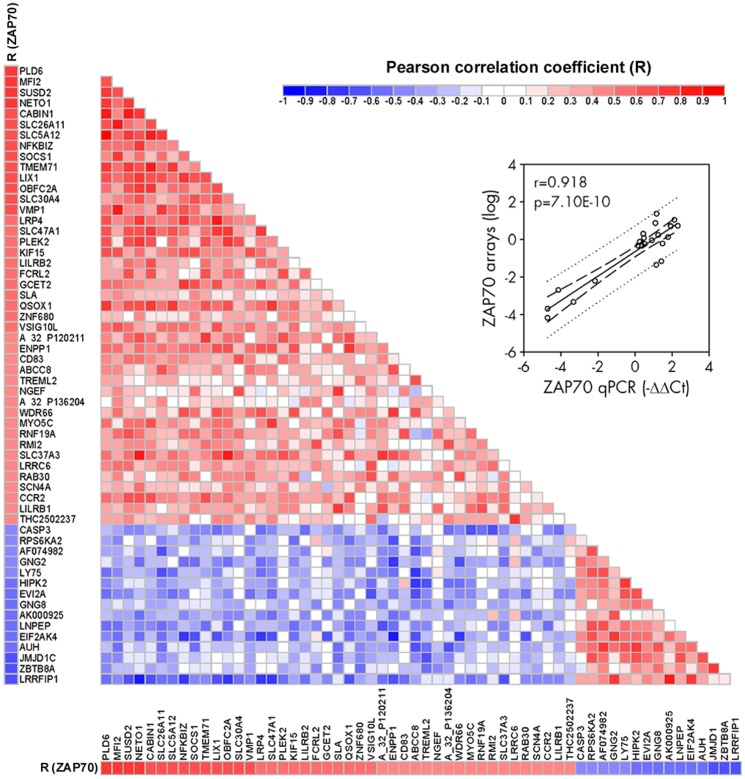
Pearson correlation analysis of the IL-4 upregulated targets compared between themselves and with ZAP-70. Triangular heat map representing the pairwise correlation coefficients (R) of the IL-4 upregulated targets between themselves. The IL-4 targets are ordered according to their correlation coefficients with ZAP-70, which are represented at the left and bottom sides. Cut-off values for positive or negative correlations with ZAP-70 were set at 0.4 and −0.4, respectively. In the event that several probes represent the same gene, only one is shown. Correlation coefficients are depicted according to the shown color scale. Inset shows Pearson correlation analysis between ZAP-70 levels by microarray (expressed as log_2_ ratios) and by qPCR (expressed as –ΔΔCt ratios).

To determine whether the gene expression response to IL-4 was homogeneous between patients, a correlation analysis between changes of every pair of genes, and between changes of each gene and the basal levels of expression of ZAP-70, CD38, and ITGA4 (whose levels did not significantly change under culture or treatment with IL-4) were performed. The numbers of non-redundant entities correlated positively or negatively with ZAP-70 were 42 and 20, respectively, setting the cut-off values of R-coefficient at ±0.4 (Figure 3; the R-coefficients for all the IL-4 targets are shown in Table S10). Expression of ZAP-70 determined by microarray and qPCR were correlated (Figure 3 inset). This analysis indicates that specific sets of genes were preferentially induced in the ZAP-70 positive or negative patients. According to this criterion, the IL-4 targets were categorized as ZAP-70^Pos^ or ZAP-70^Neg^, respectively. These conclusions were validated by hierarchical clustering analysis of these sets of genes using their fold changes, resulting in segregation of patients into two clusters, one containing the ZAP-70 positive patients and the other the ZAP-70 negative patients (Figure S3). Expression of ZAP-70, CD38, and ITGA4 significantly correlated in CLL (R = 0.512 for ZAP-70 and CD38, R = 0.509 for ZAP-70 and ITGA4, and R = 0.393 for CD38 and ITGA4); and in general, also correlated similarly with the IL-4 targets (e.g., positive correlations with SUSD2, CABIN1, OBFC2A, SLC5A12, SLC37A3, PLD6, or negative correlations with EVI2A), though the number of IL-4 targets significantly correlated with ZAP-70 was higher than with CD38 or ITGA4 (Table S10). Analysis of the gene response to IL-4 according to cytogenetic characteristics of patients or between untreated and previously treated patients did not result in significant findings, possibly due to the low number of cases within some of the groups compared. Therefore, the gene response to IL-4 is related to the expression of CLL prognostic markers, especially ZAP-70.

### Functional interpretation of the IL-4 target data sets using the MetaCore database and analysis tools

**Figure 4:**
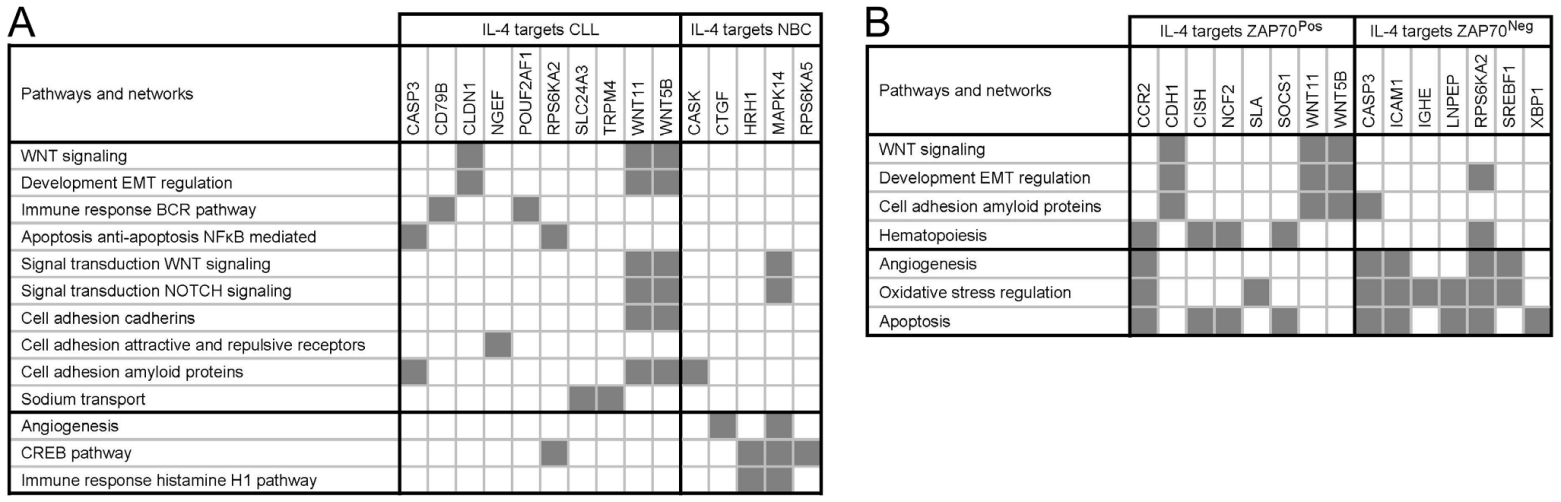
Pathways and networks differently affected according to MetaCore analysis of IL-4 targets. (A) Pathways and networks differently affected in CLL and NBC. (B) Pathways and networks differently affected in ZAP-70 positive and negative patients. Relevant genes for these functions within each group are specified.

Based on upregulation of specific genes, MetaCore analysis of the CLL data set suggested activation of Wnt signaling and cell adhesion, whereas analysis of the NBC data set suggested activation of Creb signaling and angiogenesis (Fig. 4A). Analysis of the ZAP-70^Pos^ data set suggested activation of Wnt signaling, regulation of epithelial to mesenchymal transition, and cell adhesion, whereas analysis of the ZAP-70^Neg^ data set suggested activation of oxidative stress regulation, and angiogenesis (Figure 4B). Taken together, MetaCore functional analyses support that IL-4 may transduce specific pathways in CLL and NBC, and according to ZAP-70 expression in CLL.

### Correlations between IL-4 targets and apoptosis

**Figure 5:**
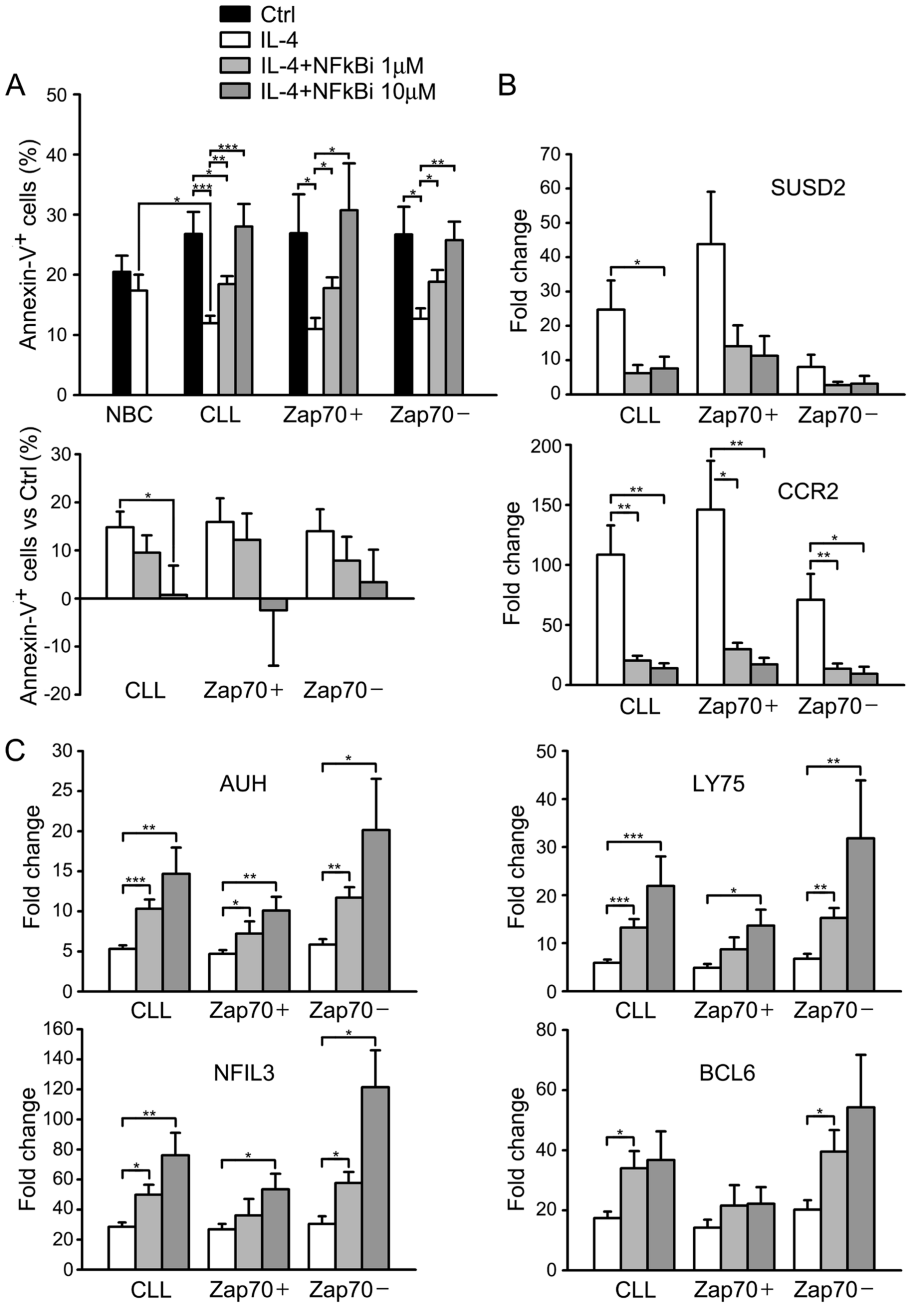
Effect of an NFκB inhibitor on apoptosis and gene expression response to IL-4 in CLL. (A) Apoptosis of NBC and CLL cells cultured for 18 h in the absence or presence of IL-4, and CLL cells cultured with IL-4 plus an NFκB activation inhibitor (NFκBi) at 1 µM and 10 µM. CLL are represented together and also separated in ZAP-70 positive and negative. Apoptosis was measured as the percentage of cells labelled with Annexin V (*top panel*). The *bottom panel* represents results in CLL patients after subtracting the percentage of apoptotic cells of the Ctrl samples to the IL-4, IL-4 plus 1 µM NFκBi, and IL-4 plus 10 µM NFκBi samples. T tests were used to compare the levels of apoptosis between cell types and conditions. When differences were significant the p-values are indicated as follows: *, p<0.05; **, p<0.01; ***, p<0.001. (B) Expression of the ZAP-70^Pos^ IL-4 targets SUSD2 and CCR2, and (C) of the ZAP-70^Neg^ IL-4 targets AUH, LY75, NFIL3, and BCL6), measured by qPCR, in 7 ZAP-70 positive and 8 ZAP-70 negative patients. The ratios for expression of IL-4 targets following treatment with IL-4 alone, IL-4 plus NFκBi at 1 µM, and IL-4 plus NFκBi at 10 µM, compared to Ctrl, are represented. P-values are depicted as in A.

Apoptosis was studied by flow cytometry analysis of annexin V positive cells. IL-4 significantly reduced spontaneous apoptosis of CLL cells, but not of NBC cells in this time period (Figure 5A). Therefore, CLL cells were more prone to spontaneous apoptosis but also better protected by IL-4 than NBC, in agreement with previous studies [13]. Differential basal expression between CLL and NBC of apoptosis-related genes, such as Bcl2 family members, and/or changes of expression during culture, might account for the different sensitivity to spontaneous apoptosis. Within the Bcl2 family, we observed significant basal overexpression of pro-apoptotic BBC3 and BMF in CLL, but also underexpression of pro-apoptotic BCL2L11/BIM (Table S2). According to MetaCore analysis, additional 86 apoptosis-related genes had different basal levels (data not shown), being some of them more than 10-fold underexpressed in CLL (GNG11, IL6, FHL2, NGFRAP1, and ITGB3). Cell culture induced changes of an elevated number of genes that persisted in the presence of IL-4 in CLL (Table S6) and in NBC (Table S7). Of them, FOS, FOSB, DUSP1, CEBPD, and ITGB2 were more than 5-fold downregulated in both cell types, GZMA and JUN had stronger downregulation in CLL (indeed, JUN was one of the IL-4 targets (Table S3)), and MYLK, PRKAR2B, SELP, FHL2, JUND, SMPD3, and CLDN5, in NBC. Within the Bcl2 family, BCLAF1 was downregulated by cell culture similarly in CLL and NBC, and BMF was upregulated by cell culture only in NBC. With regards to the differential anti-apoptotic effect of IL-4, MetaCore analysis identified, as related to apoptosis, the IL-4 upregulated targets CASP3, CCR2, CISH, GFI1, ICAM1, LNPEP, NCF2, NFKBIZ, RPS6KA2, SOCS1, and XBP1, and the IL-4 downregulated target GADD45B, in CLL (Table S3), but only CCR2, CISH, SOCS1, and XBP1, in NBC (Table S4). In addition, CAMKK1 and ESR2 were upregulated by cell culture but recovered their baseline levels by IL-4 in CLL (Table S8), and GNG4 and HRK were upregulated, and JAK2 was downregulated by cell culture, but their levels were stabilized by IL-4 in NBC (Table S9). Therefore, many genes may contribute to the increased response of CLL to IL-4. In addition, we compared the percentages of protection and the levels of change of the IL-4 targets in CLL, and found significant correlations, pointing out new potential anti-apoptotic players, of which HOMER2 and BCL6 had the highest increases (Table 2). No significant differences were observed in the levels of spontaneous apoptosis or protection by IL-4 between ZAP-70 positive and negative patients (Figure 5A), despite the fact that levels of some apoptosis-related IL-4 targets suffered significantly higher increases in ZAP-70 positive patients (SOCS1, NFKBIZ) or in ZAP-70 negative patients (LNPEP, RPS6KA2).

**Table 2:** IL-4 targets correlated with protection by IL-4 in CLL.

Gene Symbol	Fold change^†^	R-coefficient	p-value
HOMER2	18.72	0.579	3.80E-02
BCL6	7.10	0.598	3.08E-02
CLEC4A	6.65	0.657	1.47E-02
FCRL2	5.46	0.813	7.29E-04
CABIN1	4.09	0.600	3.03E-02
RMI2	3.90	0.660	1.41E-02
LILRB2	3.86	0.665	1.32E-02
FRY	3.62	0.568	4.28E-02
GIT2	2.70	0.633	2.01E-02
TMEM71	2.66	0.735	4.22E-03
BDH2	2.65	0.668	1.25E-02
PLEK2	2.59	0.579	3.80E-02
ZNF107	2.14	0.721	5.42E-03
GADD45B	-2.89	-0.564	4.48E-02

(†) Mean fold change for comparison IL-4 vs Ctrl.

### Effects of an NFκB activation inhibitor on the gene expression response to IL-4

To investigate the role of NFκB on the gene expression response to IL-4 in CLL, we used a quinazoline that inhibits the transactivation capacity of NFκB [35], in a ZAP-70 positive patient (CLL01). We found that the NFκB inhibitor preferentially downregulated the response of the ZAP-70^Pos^ genes, and upregulated the response of the ZAP-70^Neg^ genes (Table S10). The NFκB activation inhibitor counteracted the anti-apoptotic effect of IL-4 in CLL in a dose-dependent manner, especially in ZAP-70 positive patients at 10 µM (Figure 5A). The response of selected ZAP-70^Pos^ targets (SUSD2, and CCR2 (Figure 5B)), and ZAP-70^Neg^ targets (AUH, LY75, NFIL3, and BCL6 (Figure 5C) to IL-4 plus NFκB activation inhibitor was confirmed by qPCR in 7 ZAP-70 positive and 8 ZAP-70 negative patients. Upregulation of the ZAP-70^Neg^ targets by the NFκB inhibitor was higher in ZAP-70 negative patients.

## Discussion

The study of the changes in the GEPs induced by microenvironmental factors may help to understand the underlying mechanisms sustaining CLL pathogenesis. Despite the fact that IL-4 has been recognized as a key survival factor in CLL for a long time, the GEPs induced by IL-4 in CLL are poorly known. Here, we identified 229 well-defined genes as IL-4 targets in CLL and NBC altogether, most of which were novel IL-4 targets for CLL, B cells of diverse origin, lymphocytes, or other cell types. The previously known IL-4 targets that were also identified in our study provided a proof of validity for our microarray study. An additional validation was obtained by qPCR analysis on a significant set of genes. The introduction of two reference samples helped to define accurately the IL-4 targets by exclusion of those genes modulated by cell culture, but in general, comparison IL-4 vs Ctrl was necessary and sufficient.

In our analysis, the number of IL-4 targets was higher in CLL than in NBC. This outcome has two alternative (non-exclusive) explanations. First, that the different size of patient and control populations favors an increased detection of statistically significant targets with relatively heterogeneous response in CLL. Second, that indeed CLL have a stronger gene expression response. A substantial part of the gene response was common to CLL and NBC, qualitatively and quantitatively. However, sets of 54 and 11 genes with differential responses (most of them specific) were found in CLL and NBC, respectively. Previous studies had reported increased expression of the IL-4 receptor in CLL, at the protein [13], and the mRNA level by microarray [5], but in another study differences were not found [36]. In our study, as in the latter, no significant differences were observed between the basal levels of IL4R mRNA in CLL and NBC. The first two studies were performed with lower number of samples and/or used B cell samples of different source (tonsil). Furthermore, in our study the IL4R gene was induced similarly by IL-4 in CLL and NBC, suggesting that the differential response arises downstream of IL4R.

**Figure 6:**
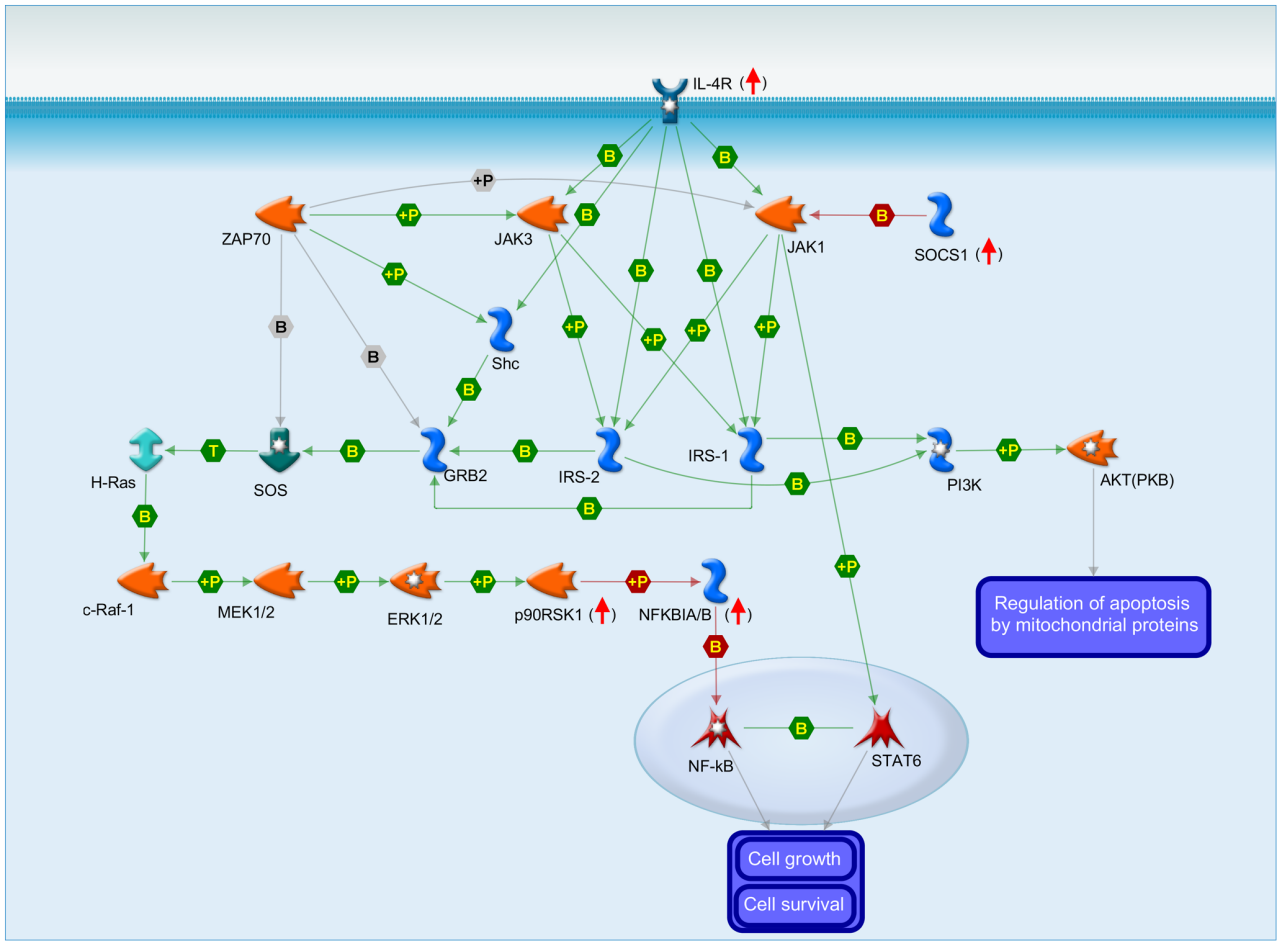
Map of the IL-4 signaling pathway and the potential role of ZAP-70. Adaptation of the pathway map entitled “Immune response_IL-4 – anti-apoptotic action” from MetaCore from Thomson Reuters. The map has been simplified leaving the minimal elements for activation of transcription factors essential in regulation of gene expression. ZAP-70 and its interactions with members of the pathway reported in the MetaCore database have been added to suggest its potential involvement in the pathway, and a link has been added for the reported interaction between NFκB and STAT6 [21]. Red arrows indicate that IL4R, SOCS1, RPS6AK2, and NFKBIZ are components of the pathway identified as IL-4 upregulated targets in this study. Other genes of the pathway were significantly regulated by cell culture, such as AKT3 (up) and GRB2 (down).

Correlations with useful prognostic markers may help to reveal new altered pathways and alternative therapeutic targets. We observed that two sets of IL-4 targets had changes correlated positively and negatively with the basal levels of ZAP-70. ZAP-70 has not been previously related to the IL-4 pathway, but several interactions with components of this pathway had been reported (Figure 6). Differentially regulated genes play essential roles in developmental and survival pathways, such as Wnt signaling and cell adhesion in the ZAP-70^Pos^ set (CDH1, WNT5B, WNT11). CDH1 expression was reported to be repressed epigenetically in CLL [37], and our data suggest that IL-4 overcomes this repression, especially in ZAP-70 positive patients (and also in patients expressing the adhesion protein ITGA4). These data may be related with recent evidence that ZAP-70 positive CLL cells exhibit higher adhesion capacity to stromal cells in response to CD40L+IL-4 [38]. Previous studies have detected increased levels of the anti-apoptotic proteins BCL2, using IL-4 [24], or MCL1, BCL2L1, BCL2A1, or XIAP, using the CD40L/IL-4 system [39], [40], without correlation at the mRNA level, and our study using IL-4 alone did not detect significant changes of expression of their transcripts either. However, our study contributes several candidate genes for the anti-apoptotic mechanism of IL-4 in CLL, for the higher sensitivity of CLL cells to cell culture, and for the higher protective effect of IL-4 in CLL cells compared to NBC. Some of the IL-4 targets, and some of the genes whose levels were altered by culture but stabilized by IL-4, were previously related to apoptosis, and their responses were often stronger in CLL (e.g. CASP3, GFI1, ICAM1, LNPEP, NCF2, NFKBIZ, RPS6KA2, GADD45B). In addition, several IL-4 targets, some of which had not been previously related to apoptosis, correlated to the levels of cytoprotection. The most upregulated genes of this list, HOMER2 and BCL6, also had significant stronger responses in CLL (HOMER2 for comparison IL-4 vs Pre, and BCL6 for comparison IL-4 vs Ctrl, Table S3). HOMER2 belongs to a family of scaffolding proteins that prevent neuronal apoptosis through PI3K and the glutamate receptor [41], and regulate T cell activation by binding to NFAT [42], but its role in B cells remains largely unknown. BCL6 is a repressor transcription factor associated to worse prognosis in CLL [43]. The identification of these genes provides a starting point for future studies aimed at defining precisely the survival mechanism of IL-4. In contrast to the study of Coscia and co-workers [23] which reports lower sensitivity to spontaneous apoptosis of CLL cells expressing poor prognosis markers, we did not observe significant differences in sensitivity of ZAP-70 positive and negative patients, despite differential regulation of several apoptosis-related IL-4 targets (SOCS1, NFKBIZ, LNPEP, RPS6KA2). However, we did observe differential cytoprotection following treatment with IL-4 and an NFκB inhibitor (see below).

NFκB and ZAP-70 are effectors of the BCR signalling pathway. NFκB expression is induced following BCR signalling, and associates to cell survival and expression of ZAP-70 in CLL [44]. ZAP-70 enhances the BCR signalling responses in CLL [45], [46]. NFκB inhibitors induce apoptosis of CLL cells, and ZAP-70 positive patients have higher NFκB activity and increased sensitivity [47], [48]. We show that an NFκB inhibitor counteracted the anti-apoptotic effect of IL-4, especially in ZAP-70 positive patients, and the gene expression response of a great part of IL-4 targets, especially the ZAP-70^Pos^ targets which, therefore, would depend on NFκB for IL-4 responsiveness. However, the inhibitor potentiated the response of a collection of genes, especially the ZAP-70^Neg^ targets, suggesting a novel mechanism by which ZAP-70 and NFκB could work together to diminish responsiveness to IL-4 of this gene set. Examples of genes of both groups were confirmed by qPCR, such as CCR2, the chemokine receptor of CCL2, involved in CLL survival [49], NFIL3, a pro-survival transcription factor in B cells [50], and BCL6. Revealing the existence of this dual mechanism has been made possible by performing transcriptome-wide studies. The search for a molecular explanation, including the role played by ZAP-70, will be the goal of future studies. JAK3 has also been targeted with promising results in CLL using the specific inhibitor PF-956980 [25]. Our preliminary data indicate that PF-956980, similarly to the NFκB inhibitor, counteracts protection by IL-4, but abrogates completely the gene expression response both of ZAP-70^Pos^ and ZAP-70^Neg^ targets (data not shown), suggesting that targeting this step, which blocks STAT6 activation, fully abolishes the pathway.

In summary, the present study identifies sets of new genes that respond differentially to IL-4 in CLL depending on ZAP-70 expression and NFκB activation, contributing to the understanding of the anti-apoptotic response to IL-4 of CLL. In the context of evaluating inhibition of the IL-4 pathway as a therapeutic strategy in CLL, several steps may be targeted. Because inhibition of NFκB counteracts cytoprotection by IL-4, and is associated to an attenuated response of a set of IL-4 targets, NFκB targeting should be further explored especially in CLL patients expressing bad prognostic markers.

## Supporting Information

Figure S1 **Comparison of baseline GEPs of CLL and NBC.** Heatmap representation of genes basally underexpressed (A) and overexpressed (B) in CLL compared to NBC, above a cut-off value of 3-fold change, and p values of less than 0.001, at time zero after purification of B cells. In the event that several probes represent the same gene, only one is shown. The relative level of gene expression is depicted according to the shown color scale.
(TIF)

Figure S2 **Validation of microarray analysis by qPCR.** Pearson correlation analysis between microarray and qPCR for 16 IL-4 targets. Microarray data were expressed as log fold change ratios, and qPCR data as −ΔΔCt ratios. The CLL and NBC samples are represented together. Correlation coefficients (r) and p-values are indicated. The IL-4 targets were ordered alphabetically.
(TIF)

Figure S3 **Hierarchical clustering analysis for IL-4 upregulated targets correlated with ZAP70.** (A) Hierarchical clustering analysis using the ZAP70^Pos^ IL-4 targets. (B) Hierarchical clustering analysis using the ZAP70^Neg^ IL-4 targets. In order to make groups of similar size, we selected, among the 23 patients studied by microarray, the 5 patients with the lowest levels of ZAP70, the 5 patients with the highest levels of ZAP70 and other 6 patients with intermediate values. Both analyses were able to separate CLL into two clusters containing the 5 positive and 5 negative patients. In the event that several probes represent the same gene, only one is shown. Fold changes for the IL-4 targets, or relative levels for basal expression of ZAP70 by microarray, are depicted according to the shown log_2_ color scale.
(TIF)

Table S1 **Characteristics of CLL patients.**
(XLS)

Table S2 **Differential basal gene expression levels between CLL and NBC.**
(XLS)

Table S3 **IL-4 targets in CLL and comparison of their responses with those in NBC.**
(XLS)

Table S4 **IL-4 targets in NBC and comparison of their responses with those in CLL.**
(XLS)

Table S5 **Novel and previously known IL-4 targets in diverse cell types (with references) among the IL-4 targets identified in CLL and NBC in the present study.**
(XLS)

Table S6 **Gene expression changes induced by culture not counteracted by IL-4 in CLL.**
(XLS)

Table S7 **Gene expression changes induced by culture not counteracted by IL-4 in NBC.**
(XLS)

Table S8 **Gene expression changes induced by culture counteracted by IL-4 in CLL.**
(XLS)

Table S9 **Genes expression changes induced by culture counteracted by IL-4 in NBC.**
(XLS)

Table S10 **Effects of an NFκB activation inhibitor on the response of the IL-4 upregulated targets in a CLL patient (CLL01, Zap70 positive).**
(XLS)

## References

[1] Chiorazzi N (2012) Implications of new prognostic markers in chronic lymphocytic leukemia. Hematology Am Soc Hematol Educ Program 2012: 76–87. 23233564 10.1182/asheducation-2012.1.76

[2] Klein U, Tu Y, Stolovitzky GA, Mattioli M, Cattoretti G, et al (2001) Gene expression profiling of B cell chronic lymphocytic leukemia reveals a homogeneous phenotype related to memory B cells. J Exp Med 194: 1625–1638. 11733577 10.1084/jem.194.11.1625 PMC2193527

[3] Rosenwald A, Alizadeh AA, Widhopf G, Simon R, Davis RE, et al (2001) Relation of gene expression phenotype to immunoglobulin mutation genotype in B cell chronic lymphocytic leukemia. J Exp Med 194: 1639–1647. 11733578 10.1084/jem.194.11.1639 PMC2193523

[4] Stratowa C, Löffler G, Lichter P, Stilgenbauer S, Haberl P, et al (2001) CDNA microarray gene expression analysis of B-cell chronic lymphocytic leukemia proposes potential new prognostic markers involved in lymphocyte trafficking. Int J Cancer 91: 474–480. 11251968 10.1002/1097-0215(200002)9999:9999<::aid-ijc1078>3.0.co;2-c

[5] Zheng Z, Venkatapathy S, Rao G, Harrington CA (2002) Expression profiling of B cell chronic lymphocytic leukemia suggests deficient CD1-mediated immunity, polarized cytokine response, altered adhesion and increased intracellular protein transport and processing of leukemic cells. Leukemia 16: 2429–2437. 12454749 10.1038/sj.leu.2402711

[6] Jelinek DF, Tschumper RC, Stolovitzky GA, Iturria SJ, Tu Y, et al (2003) Identification of a global gene expression signature of B-chronic lymphocytic leukemia. Mol Cancer Res 1: 346–361. 12651908

[7] Wang J, Coombes KR, Highsmith WE, Keating MJ, Abruzzo LV (2004) Differences in gene expression between B-cell chronic lymphocytic leukemia and normal B cells: a meta-analysis of three microarray studies. Bioinformatics 20: 3166–3178. 15231529 10.1093/bioinformatics/bth381

[8] Dürig J, Nückel H, Hüttmann A, Kruse E, Hölter T, et al (2003) Expression of ribosomal and translation-associated genes is correlated with a favorable clinical course in chronic lymphocytic leukemia. Blood 101: 2748–2755. 12456497 10.1182/blood-2002-09-2683

[9] Wiestner A, Rosenwald A, Barry TS, Wright G, Davis RE, et al (2003) ZAP-70 expression identifies a chronic lymphocytic leukemia subtype with unmutated immunoglobulin genes, inferior clinical outcome, and distinct gene expression profile. Blood 101: 4944–4951. 12595313 10.1182/blood-2002-10-3306

[10] Stankovic T, Hubank M, Cronin D, Stewart GS, Fletcher D, et al (2004) Microarray analysis reveals that TP53- and ATM-mutant B-CLLs share a defect in activating proapoptotic responses after DNA damage but are distinguished by major differences in activating prosurvival responses. Blood 103: 291–300. 12958068 10.1182/blood-2003-04-1161

[11] Rodríguez A, Villuendas R, Yáñez L, Gómez ME, Díaz R, et al (2007) Molecular heterogeneity in chronic lymphocytic leukemia is dependent on BCR signaling: clinical correlation. Leukemia 21: 1984–1991. 17611561 10.1038/sj.leu.2404831

[12] Seifert M, Sellmann L, Bloehdorn J, Wein F, Stilgenbauer S, et al (2012) Cellular origin and pathophysiology of chronic lymphocytic leukemia. J Exp Med 209: 2183–2198. 23091163 10.1084/jem.20120833 PMC3501361

[13] Douglas RS, Capocasale RJ, Lamb RJ, Nowell PC, Moore JS (1997) Chronic lymphocytic leukemia B cells are resistant to the apoptotic effects of Transforming Growth Factor-β. Blood 89: 941–947. 9028325

[14] Burger JA, Montserrat E (2013) Coming full circle: 70 years of chronic lymphocytic leukemia cell redistribution, from glucocorticoids to inhibitors of B-cell receptor signaling. Blood 121: 1501–1509. 23264597 10.1182/blood-2012-08-452607 PMC4968370

[15] Okada H, Banchereau J, Lotze MT (2003) Interleukin-4. In Thompson AW, Lotze MT, editors. The Cytokine Handbook, Vol. I . London: Academic Press. pp 227–262.

[16] Zamorano J, Mora AL, Boothby M, Keegan AD (2001) NF-kappa B activation plays an important role in the IL-4-induced protection from apoptosis. Int Immunol 13: 1479–1487. 11717189 10.1093/intimm/13.12.1479

[17] Furman RR, Asgary Z, Mascarenhas JO, Liou HC, Schattner EJ (2000) Modulation of NF-kappa B activity and apoptosis in chronic lymphocytic leukemia B cells. J Immunol 164: 2200–2206. 10657675 10.4049/jimmunol.164.4.2200

[18] Cuní S, Pérez-Aciego P, Pérez-Chacón G, Vargas JA, Sánchez A, et al (2004) A sustained activation of PI3K/NF-kappaB pathway is critical for the survival of chronic lymphocytic leukemia B cells. Leukemia 18: 1391–1400. 15175625 10.1038/sj.leu.2403398

[19] Thieu VT, Nguyen ET, McCarthy BP, Bruns HA, Kapur R, et al (2007) IL-4-stimulated NF-kappaB activity is required for Stat6 DNA binding. J Leukoc Biol 82: 370–379. 17513694 10.1189/jlb.1106707

[20] Bhattacharya N, Reichenzeller M, Caudron-Herger M, Haebe S, Brady N, et al.. (2014) Loss of cooperativity of secreted CD40L and increased dose-response to IL4 on CLL cell viability correlates with enhanced activation of NF-kB and STAT6. Int J Cancer. doi: 10.1002/ijc.28974. [Epub ahead of print]. 10.1002/ijc.28974 24828787

[21] Shen CH, Stavnezer J (1998) Interaction of stat6 and NF-kappaB: direct association and synergistic activation of interleukin-4-induced transcription. Mol Cell Biol 18: 3395–3404. 9584180 10.1128/mcb.18.6.3395 PMC108921

[22] Messner B, Stütz AM, Albrecht B, Peiritsch S, Woisetschläger M (1997) Cooperation of binding sites for STAT6 and NF kappa B/rel in the IL-4-induced up-regulation of the human IgE germline promoter. J Immunol 159: 3330–3337. 9317131

[23] Coscia M, Pantaleoni F, Riganti C, Vitale C, Rigoni M, et al (2011) IGHV unmutated CLL B cells are more prone to spontaneous apoptosis and subject to environmental prosurvival signals than mutated CLL B cells. Leukemia 25: 828–837. 21372840 10.1038/leu.2011.12

[24] Dancescu M, Rubio-Trujillo M, Biron G, Bron D, Delespesse G, et al (1992) Interleukin 4 protects chronic lymphocytic leukemic B cells from death by apoptosis and upregulates Bcl-2 expression. J Exp Med 176: 1319–1326. 1402678 10.1084/jem.176.5.1319 PMC2119420

[25] Steele AJ, Prentice AG, Cwynarski K, Hoffbrand AV, Hart SM, et al (2010) The JAK3-selective inhibitor PF-956980 reverses the resistance to cytotoxic agents induced by interleukin-4 treatment of chronic lymphocytic leukemia cells: potential for reversal of cytoprotection by the microenvironment. Blood 116: 4569–4577. 20716767 10.1182/blood-2009-09-245811

[26] Mainou-Fowler T, Proctor SJ, Miller S, Dickinson AM (2001) Expression and production of interleukin 4 in B-cell chronic lymphocytic leukaemia. Leuk Lymphoma 42: 689–698. 11697499 10.3109/10428190109099331

[27] Pangault C, Amé-Thomas P, Ruminy P, Rossille D, Caron G, et al (2010) Follicular lymphoma cell niche: identification of a preeminent IL-4-dependent T(FH)–B cell axis. Leukemia 24: 2080–2089. 20944673 10.1038/leu.2010.223 PMC3317889

[28] Schroder AJ, Pavlidis P, Arimura A, Capece D, Rothman PB (2002) Cutting edge: STAT6 serves as a positive and negative regulator of gene expression in IL-4-stimulated B lymphocytes. J Immunol 168: 996–1000. 11801631 10.4049/jimmunol.168.3.996

[29] Lu X, Nechushtan H, Ding F, Rosado MF, Singal R, et al (2005) Distinct IL-4-induced gene expression, proliferation, and intracellular signaling in germinal center B-cell-like and activated B-cell-like diffuse large-cell lymphomas. Blood 105: 2924–2932. 15591113 10.1182/blood-2004-10-3820

[30] Stamatopoulos B, Meuleman N, De Bruyn C, Pieters K, Anthoine G, et al (2010) A molecular score by quantitative PCR as a new prognostic tool at diagnosis for chronic lymphocytic leukemia patients. PLoS ONE 5: 12780. 10.1371/journal.pone.0012780 PMC2940823 20862275

[31] Early SB, Barekzi E, Negri J, Hise K, Borish L, et al (2007) Concordant modulation of cysteinyl leukotriene receptor expression by IL-4 and IFN-gamma on peripheral immune cells. Am J Respir Cell Mol Biol 36: 715–720. 17272825 10.1165/rcmb.2006-0252OC PMC2720145

[32] Finkelman FD, Katona IM, Urban JF Jr, Snapper CM, Ohara J, et al (1986) Suppression of in vivo polyclonal IgE responses by monoclonal antibody to the lymphokine B-cell stimulatory factor 1. Proc Natl Acad Sci USA 83: 9675–9678. 3491987 10.1073/pnas.83.24.9675 PMC387203

[33] Kashiwada M, Levy DM, McKeag L, Murray K, Schröder AJ, et al (2010) IL-4-induced transcription factor NFIL3/E4BP4 controls IgE class switching. Proc Natl Acad Sci USA 107: 821–826. 20080759 10.1073/pnas.0909235107 PMC2818942

[34] Alcaraz-García MJ, Ruiz-Lafuente N, Sebastián-Ruiz S, Majado MJ, González-García C, et al (2011) Human and mouse DOCK10 splicing isoforms with alternative first coding exon usage are differentially expressed in T and B lymphocytes. Hum Immunol 72: 531–537. 21514340 10.1016/j.humimm.2011.03.024

[35] Tobe M, Isobe Y, Tomizawa H, Nagasaki T, Takahashi H, et al (2003) A novel structural class of potent inhibitors of NF-kappa B activation: structure-activity relationships and biological effects of 6-aminoquinazoline derivatives. Bioorg Med Chem 11: 3869–3878. 12927847 10.1016/s0968-0896(03)00438-3

[36] Kaminski A, Demaine A, Prentice A (1998) Cytoplasmic interleukin-4 (IL-4) and surface IL-4 receptor expression in patients with B-cell lymphocytic leukemia. Blood 92: 2188–2189. 9731083

[37] Moskalev EA, Luckert K, Vorobjev IA, Mastitsky SE, Gladkikh AA, et al (2012) Concurrent epigenetic silencing of wnt/β-catenin pathway inhibitor genes in B cell chronic lymphocytic leukaemia. BMC Cancer 2012 12: 213. 10.1186/1471-2407-12-213 PMC3489542 22672427

[38] Lafarge ST, Johnston JB, Gibson SB, Marshall AJ (2014) Adhesion of ZAP-70+ chronic lymphocytic leukemia cells to stromal cells is enhanced by cytokines and blocked by inhibitors of the PI3-kinase pathway. Leuk Res 38: 109–115. 23981382 10.1016/j.leukres.2013.08.001

[39] Willimott S, Baou M, Naresh K, Wagner SD (2007) CD154 induces a switch in pro-survival Bcl-2 family members in chronic lymphocytic leukaemia. Br J Haematol 138: 721–732. 17760804 10.1111/j.1365-2141.2007.06717.x

[40] Cosimo E, McCaig AM, Carter-Brzezinski LJM, Wheadon H, Leach MT, et al (2013) Inhibition of NF-kB-mediated signaling by the cyclin-dependent kinase inhibitor CR8 overcomes prosurvival stimuli to induce apoptosis in chronic lymphocytic leukemia cells. Clin Cancer Res 19: 2393–2405. 23532892 10.1158/1078-0432.CCR-12-2170

[41] Rong R, Ahn JY, Huang H, Nagata E, Kalman D, et al (2003) PI3 kinase enhancer-Homer complex couples mGluRI to PI3 kinase, preventing neuronal apoptosis. Nat Neurosci 6: 1153–1161. 14528310 10.1038/nn1134

[42] Huang GN, Huso DL, Bouyain S, Tu J, McCorkell KA, et al (2008) NFAT binding and regulation of T cell activation by the cytoplasmic scaffolding Homer proteins. Science 319: 476–481. 18218901 10.1126/science.1151227 PMC3602998

[43] Jantus Lewintre E, Reinoso Martín C, García Ballesteros C, Pendas J, Benet Campos C, et al (2009) BCL6: somatic mutations and expression in early-stage chronic lymphocytic leukemia. Leuk Lymphoma 50: 773–780. 19367498 10.1080/10428190902842626

[44] López-Guerra M, Roué G, Pérez-Galán P, Alonso R, Villamor N, et al (2009) p65 activity and ZAP-70 status predict the sensitivity of chronic lymphocytic leukemia cells to the selective IkappaB kinase inhibitor BMS-345541. Clin Cancer Res 15: 2767–2776. 19351760 10.1158/1078-0432.CCR-08-2382

[45] Chen L, Huynh L, Apgar J, Tang L, Rassenti L, et al (2008) ZAP-70 enhances IgM signaling independent of its kinase activity in chronic lymphocytic leukemia. Blood 111: 2685–2692. 18048647 10.1182/blood-2006-12-062265 PMC2254551

[46] Calpe E, Codony C, Baptista MJ, Abrisqueta P, Carpio C, et al (2011) ZAP-70 enhances migration of malignant B lymphocytes toward CCL21 by inducing CCR7 expression via IgM-ERK1/2 activation. Blood 118: 4401–4410. 21865343 10.1182/blood-2011-01-333682

[47] Pickering BM, de Mel S, Lee M, Howell M, Habens F, et al (2007) Pharmacological inhibitors of NF-kappaB accelerate apoptosis in chronic lymphocytic leukaemia cells. Oncogene 26: 1166–1177. 16924235 10.1038/sj.onc.1209897

[48] Hewamana S, Alghazal S, Lin TT, Clement M, Jenkins C, et al (2008) The NF-kappaB subunit Rel A is associated with in vitro survival and clinical disease progression in chronic lymphocytic leukemia and represents a promising therapeutic target. Blood 111: 4681–4689. 18227347 10.1182/blood-2007-11-125278

[49] Burgess M, Cheung C, Chambers L, Ravindranath K, Minhas G, et al (2012) CCL2 and CXCL2 enhance survival of primary chronic lymphocytic leukemia cells in vitro. Leuk Lymphoma 53: 1988–1998. 22397722 10.3109/10428194.2012.672735

[50] Ikushima S, Inukai T, Inaba T, Nimer SD, Cleveland JL, et al (1997) Pivotal role for the NFIL3/E4BP4 transcription factor in interleukin 3-mediated survival of pro-B lymphocytes. Proc Natl Acad Sci U S A 94: 2609–2614. 9122243 10.1073/pnas.94.6.2609 PMC20136

