# Supplementary figures and images for: The Gene Expression Response of Chronic Lymphocytic Leukemia Cells to IL-4 Is Specific, Depends on ZAP-70 Status and Is Differentially Affected by an NFκB Inhibitor
Source: PLoS One. 2014 Oct 3;9(10):e109533. doi: 10.1371/journal.pone.0109533 (PMC4184842; doi:10.1371/journal.pone.0109533)

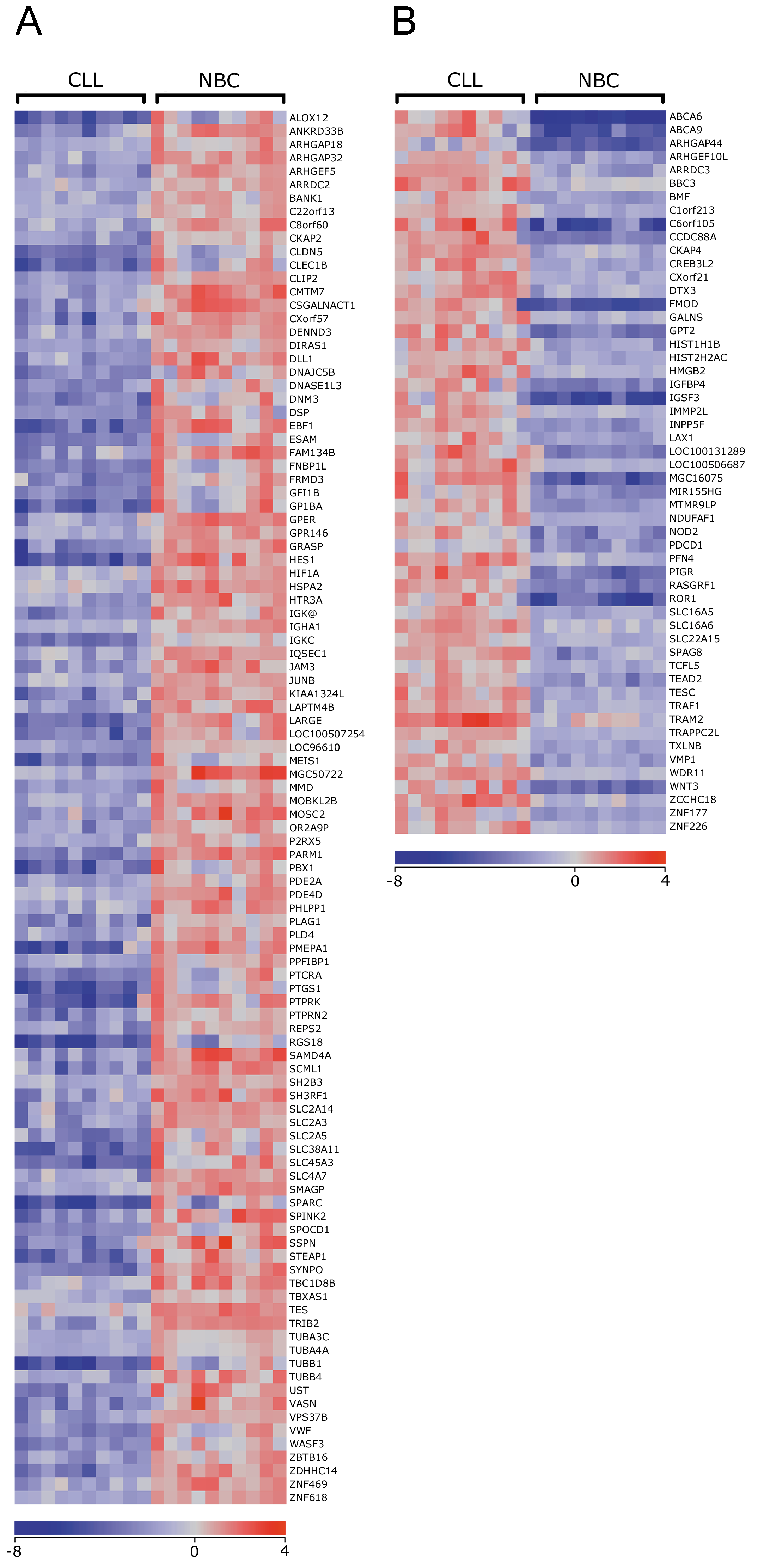

Supplement: Figure S1 — Comparison of baseline GEPs of CLL and NBC. Heatmap representation of genes basally underexpressed (A) and overexpressed (B) in CLL compared to NBC, above a cut-off value of 3-fold change, and p values of less than 0.001, at time zero after purification of B cells. In the event that several probes represent the same gene, only one is shown. The relative level of gene expression is depicted according to the shown color scale. (TIF) [file pone.0109533.s001.tif]

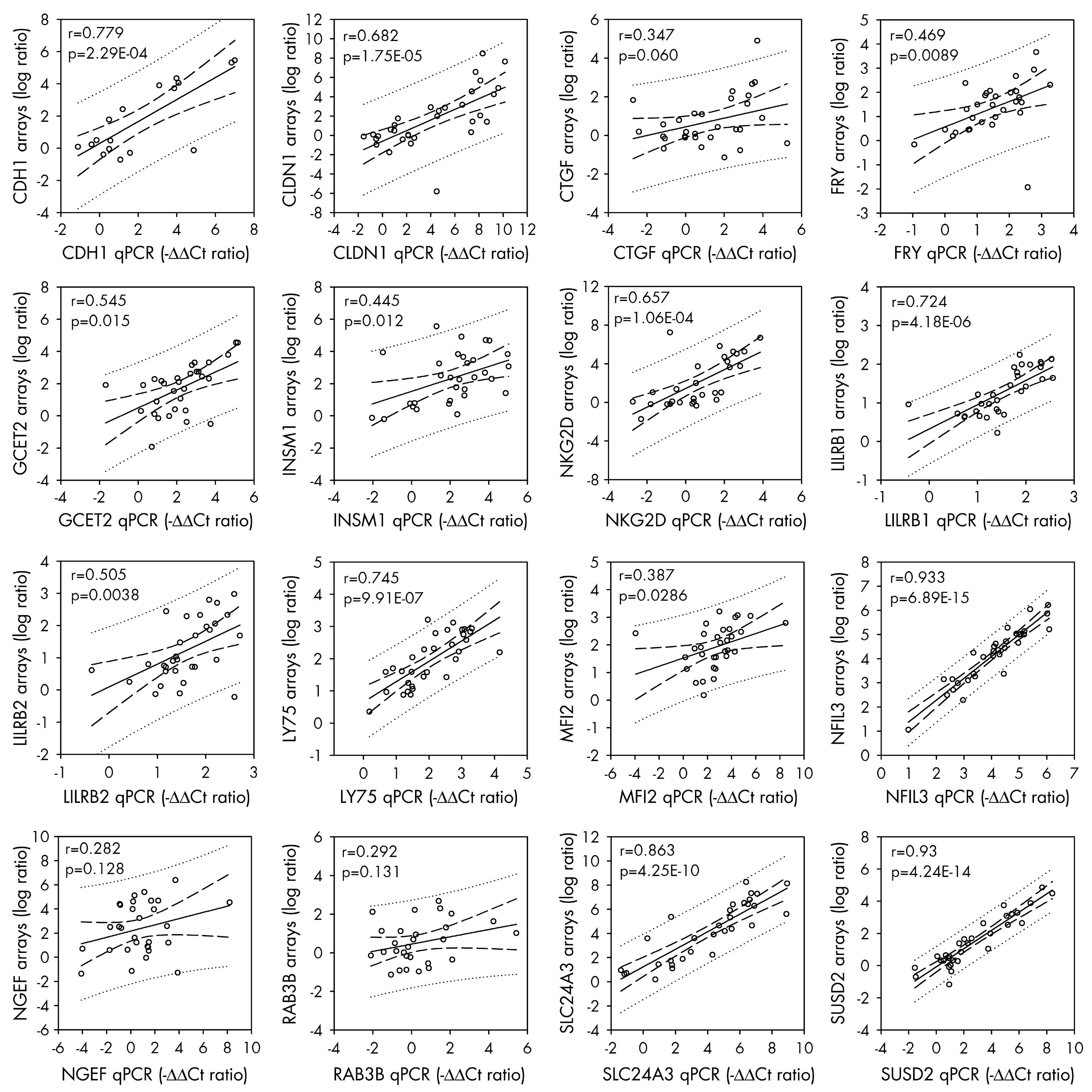

Supplement: Figure S2 — Validation of microarray analysis by qPCR. Pearson correlation analysis between microarray and qPCR for 16 IL-4 targets. Microarray data were expressed as log fold change ratios, and qPCR data as −ΔΔCt ratios. The CLL and NBC samples are represented together. Correlation coefficients (r) and p-values are indicated. The IL-4 targets were ordered alphabetically. (TIF) [file pone.0109533.s002.tif]

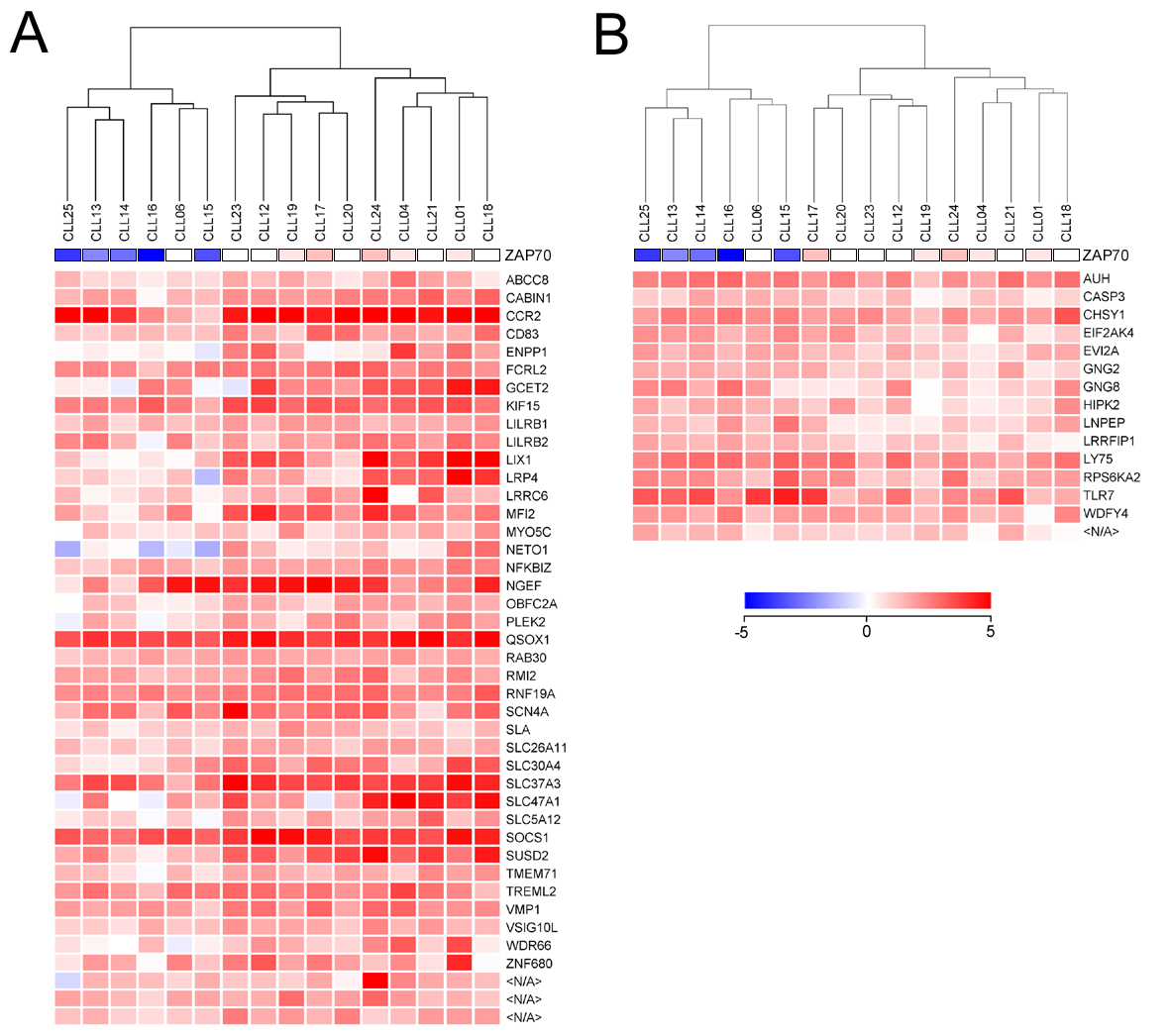

Supplement: Figure S3 — Hierarchical clustering analysis for IL-4 upregulated targets correlated with ZAP70. (A) Hierarchical clustering analysis using the ZAP70Pos IL-4 targets. (B) Hierarchical clustering analysis using the ZAP70Neg IL-4 targets. In order to make groups of similar size, we selected, among the 23 patients studied by microarray, the 5 patients with the lowest levels of ZAP70, the 5 patients with the highest levels of ZAP70 and other 6 patients with intermediate values. Both analyses were able to separate CLL into two clusters containing the 5 positive and 5 negative patients. In the event that several probes represent the same gene, only one is shown. Fold changes for the IL-4 targets, or relative levels for basal expression of ZAP70 by microarray, are depicted according to the shown log2 color scale. (TIF) [file pone.0109533.s003.tif]
